# Comprehensive microbiome and metabolome analysis revealed the changes of semen microbial characteristics and metabolic phenotypes in patients with idiopathic oligoasthenozoospermia

**DOI:** 10.3389/fcimb.2025.1741184

**Published:** 2026-01-14

**Authors:** Shikuan Lu, Zhiyu Wu, Yipeng Zhao, Tang Tang, Ye Dong, Meilin Wu, Peihai Zhang, Ziyang Ma

**Affiliations:** 1 Hospital of Chengdu University of Traditional Chinese Medicine, Chengdu, China; 2 Sichuan Second Hospital of Traditional Chinese Medicine, Chengdu, China; 3 Chengdu Polytechnic, Chengdu, China

**Keywords:** idiopathic oligoasthenozoospermia, inflammation, metabolome, oxidative stress, precision medicine, semen microbiome

## Abstract

**Background:**

The etiology and pathogenesis of idiopathic oligoasthenospermia (IOA) remain unclear, and current treatment options yield suboptimal outcomes. Consequently, there is an urgent need to identify novel biomarkers and develop diagnostic tools to improve patient identification and clinical management. Multi-omics technologies offer a promising pathway toward achieving this goal in the future.

**Methods:**

This study included 40 untreated patients with idiopathic oligoasthenospermia (IOA) and 30 healthy fertile males (HP) as controls. Semen samples were analyzed using 16S rRNA gene sequencing (microbiome) and non-targeted metabolomics (GC-MS/LC-MS coupled). A microbe-metabolite association network was integrated at the genus level based on Spearman correlation algorithms.

**Results:**

Semen microbiome analysis revealed that both microbial composition and species richness differed between IOA patients and HP controls. Non-targeted metabolomics further demonstrated characteristic metabolic dysregulation in seminal plasma of IOA patients, with a metabolic signature effectively distinguishing cases from controls (VIP > 1, FDR < 0.05). KEGG pathway enrichment analysis indicated that differentially expressed metabolites primarily involved amino acid metabolism, carbohydrate metabolism, and related signaling pathways (corrected p-value < 0.05). Construction of a Spearman correlation network between microbiota and metabolites (|r| > 0.6) identified significant interactions between core bacterial genera such as *Dialister*, *Prevotellaceae_NK3B31_group*, *Lawsonella*, and *Blautia* with seminal plasma metabolites, suggesting potential involvement of the microbiota-metabolite axis in the pathological process of IOA.

**Conclusion:**

The microbial community structure and metabolic profiles in the semen of IOA patients exhibit significant disruption. Diagnostic models constructed based on combined microbial-metabolite features demonstrate potential for effectively distinguishing disease phenotypes. The core dysregulated bacterial genera, associated metabolites, and related pathways may serve as early diagnostic biomarkers and therapeutic intervention targets.

## 1 Introduction

Infertility is a global issue. The World Health Organization estimates that approximately one in six people of reproductive age worldwide experience infertility during their lifetime, with about half of these cases attributed to male factors (World Health Organization, 2023). Oligoasthenospermia (OA), a collective term for oligospermia and asthenospermia, is a major cause of male infertility. Clinically, it is primarily characterized by reduced sperm concentration and motility (Agarwal et al., 2021). Previous studies indicate that OA development is closely associated with genital infections, genital trauma, endocrine disorders, varicocele, cryptorchidism, medications, and toxins (Alahmar, 2022). However, 30%-40% of male infertility cases remain idiopathic, leading to suboptimal treatment selection and outcomes (Bracke et al., 2018). The underlying mechanisms and etiology of IOA remain to be elucidated. In the era of precision medicine, identifying novel non-invasive biomarkers and developing more accurate diagnostic tools to recognize IOA in clinical settings is crucial. These unknown molecular biomarkers not only facilitate etiological diagnosis for IOA patients but also provide valuable insights for developing targeted therapies for this conditio (Assidi, 2022).

Component analysis has long been employed in diagnosing male infertility. Given the relative ease of obtaining semen samples and their provision of routine parameters regarding macroscopic characteristics such as sperm morphology, count, and motility, semen serves as a prospective source of biomarkers for infertility. In the post-genomic era, omics technologies—including metagenomics, proteomics, metabolomics, transcriptomics, immunomics, and multi-omics integration—can characterize proteins, genes, metabolites, and epigenetic features associated with male infertility, offering potential for elucidating the molecular and pathophysiological mechanisms of IOA (Llavanera et al., 2022). Advancements in microbiome technologies, such as bioinformatics and high-throughput sequencing, enable the study of all environmental microorganisms without reliance on cultivation. Through the microbiome, researchers can identify compositional differences in microbial communities and further predict or interpret functional alterations in these communities (Manos, 2022). The metabolome directly reflects the functional interactions between microbiota and the host. The combined analysis of powerful tools from microbiomics and metabolomics reveals how microbial communities shape the metabolic state of the host or environment through their metabolic activities and co-metabolism with the host, offering new perspectives and approaches for disease prevention, diagnosis, and treatment (Shaffer et al., 2017).

To date, few studies have explored the seminal microbiome and its metabolite alterations associated with IOA patients. Therefore, we addressed this critical gap in the literature by We employed 16S rRNA sequencing and non-targeted metabolomics to conduct a comprehensive, systematic investigation of the microbiome and metabolome in human semen samples. This approach aimed to determine variations in semen microbial diversity and metabolite abundance, decipher the association between IOA and semen microbiota/metabolites, and enhance our understanding of how semen microbial communities influence the host through metabolic activities. Ultimately, this research seeks to develop more accurate diagnostic tools and effective therapeutic strategies.

## 2 Materials and methods

### 2.1 Subject recruitment

A total of 40 patients diagnosed with IOA and 30 healthy subjects were recruited between March 2023 and January 2024 at Chengdu University of Traditional Chinese Medicine Affiliated Hospital (Chengdu, China) following comprehensive systematic evaluation. All participants voluntarily signed informed consent forms to participate in this study. Healthy male subjects whose partners had given birth or become pregnant within the previous year and whose current semen test results were normal.IOA patients first met the WHO criteria for oligoasthenozoospermia diagnosis, with additional inclusion criteria as follows: (1) No history of genital tract infection or inflammation within the past 3 months; (2) Negative bacterial culture, mycoplasma, and chlamydia testing in semen; (3) Absence of organic lesions in the genitourinary system; (4) No use of antibiotics or related biological products within the past month; (5) No systemic diseases. (6)Without genetic factors, such as chromosomal abnormalities.

### 2.2 Ethical approval

All participants signed the informed consent form before inclusion. This study was approved by the Ethics Committee of the Affiliated Hospital of Chengdu University of Traditional Chinese Medicine (Chengdu, China) (Ethics Approval Number: 2024KL-016).

### 2.3 Sample collection

Semen samples were collected *via* masturbation at the Andrology Laboratory of Chengdu University of Traditional Chinese Medicine Affiliated Hospital (Chengdu, China) and stored in sterile glass containers following a 3- to 5-day abstinence period. The semen sample was obtained through masturbation under a sterile condition.Samples were stored in a -80°C freezer within 2 hours of collection.

### 2.4 16S diversity sequencing

#### 2.4.1 Semen DNA extraction and PCR amplification

Genomic DNA was extracted from semen samples using the MagPure Soil DNA LQ Kit (Magan) according to the manufacturer's instructions. DNA concentration and purity were assessed using NanoDrop 2000 (Thermo Fisher Scientific, USA) and agarose gel electrophoresis. Extracted DNA was stored at -20°C. Using barcoded specific primers and Takara Ex Taq High Fidelity Enzyme, PCR amplification of bacterial 16S rRNA genes was performed with the extracted genomic DNA as template. The V3-V4 variable region of the 16S rRNA gene was amplified using universal primers 343F (5'-TACGGRAGGCAGCAG-3') and 798R (5'-AGGGTATCTAATCCT-3') (Nossa et al., 2010)for bacterial diversity analysis.

#### 2.4.2 Library construction and sequencing

PCR amplification products were detected by agarose gel electrophoresis. They were then purified using AMPure XP magnetic beads. The purified products served as templates for second-round PCR amplification. Following a second round of PCR amplification, the products were purified again using magnetic beads. The purified second-round products underwent Qubit quantification, and their concentration was adjusted for sequencing. Sequencing was performed using the Illumina NovaSeq 6000 platform, generating 250 bp paired-end reads. Sequencing services were provided by Shanghai Ouyi Biotechnology Co., Ltd. (Shanghai, China).

#### 2.4.3 Bioinformatics analysis

Library preparation, sequencing, and data analysis were performed by Shanghai Ouyi Biomedical Technology Co., Ltd. Raw data were in FASTQ format. After data download, Cutadapt software was first used to trim primer sequences from raw data sequences. Subsequently, DADA2 (Callahan et al., 2016)was employed to perform quality filtering, denoising, assembly, and de-chimerization on the qualified paired-end raw data using default parameters of QIIME 2 (Bolyen et al., 2019), yielding representative sequences and abundance tables for amplicon sequences (AS). After selecting representative sequences for each ASV using the QIIME 2 software package, all representative sequences were annotated by aligning them against the Silva database (version 138). Species annotation was performed using the q2-feature-classifier software with default parameters. QIIME 2 software was employed for α and β diversity analysis. Alpha diversity was assessed using indices including Chao1 (Chao and Bunge, 2002) and ACE. Principal coordinate analysis (PCoA) was performed using a binary Jaccard distance matrix computed in R to assess beta diversity among samples. Differential analysis was conducted using ANOVA and Kruskal-Wallis statistical tests based on R packages. Differential analysis of species abundance profiles was performed using LEfSe.

### 2.5 Non-targeted metabolomics analysis

#### 2.5.1 Sample preparation and chromatography-mass spectrometry analysis

Metabolite extraction and detection were performed by Shanghai Luming Biotechnology Co., Ltd. (Shanghai, China) using liquid chromatography-mass spectrometry (LC-MS) and gas chromatography-mass spectrometry (GC-MS) platforms for comprehensive metabolomics analysis.

#### 2.5.2 Data preprocessing and analysis

##### 2.5.2.1 GC-MS

The raw GC-MS data (.D format) is converted into the AnalysisBaseFileConverter software's abf format for rapid data retrieval. It is then imported into MS-DIAL software for preprocessing, where algorithms extract “model peaks” from the chromatogram, remove background noise, and achieve compound identification and quantification by matching retention times, fragment ion mass spectra, and similarity scores against the custom database built by Luming Bio. MS-DIAL processes imported data through peak detection, peak identification, deconvolution, qualitative analysis, peak alignment, filtering, and missing value interpolation, ultimately exporting the raw data matrix. Within each sample, all peak signal intensities are segmented and normalized based on internal standards with RSD > 0.1 after screening. Following normalization, redundant data is removed and peaks are merged to generate the final data matrix.

##### 2.5.2.2 LC-MS

Raw LC-MS data were processed using Progenesis QI V2.3 (Nonlinear Dynamics, Newcastle, UK) for baseline filtering, peak identification, integration, retention time correction, peak alignment, and normalization. Compounds were identified using The Human Metabolome Database (HMDB), Lipidmaps (V2.3), Metlin, and LuMing Bio's in-house database based on precise mass-to-charge ratios (M/z), secondary fragments, and isotope distribution. The extracted data underwent further processing: any peaks with over 50% missing values (ion intensity=0) within a group were removed; zero values were replaced with half the minimum value; and data were filtered based on compound qualitative results. Compounds with database match scores below 36 points (out of 80 total points) were also deemed inaccurate and deleted. Positive and negative ion data were combined into a single data matrix.

Import the data matrix into the R package for principal component analysis (PCA) to observe the overall distribution among samples and the stability of the entire analysis process. Orthogonal partial least squares discriminant analysis (OPLS-DA) was employed to distinguish metabolite differences between groups. To prevent overfitting, seven-fold cross-validation and 200 response permutation tests (RPT) were used to assess model quality. Variable Importance Projection (VIP) values obtained from the OPLS-DA model were used to rank each variable's overall contribution to group discrimination. A two-tailed Student's t-test was further applied to validate whether metabolic differences between groups were statistically significant. Differential metabolites with VIP values >1.0 and p-values <0.05 were selected. The KEGG database was consulted to investigate the functions and metabolic pathways of these metabolites. Additionally, metabolic pathway enrichment analysis was performed for the differential metabolites; pathways with p-values <0.05 were considered significantly enriched.

#### 2.5.3 Statistical analysis

This study employed IBM SPSS Statistics 27 for data analysis. Measurement data followed a normal distribution and were expressed as mean ± standard deviation. Group comparisons were conducted using t-tests and one-way analysis of variance (ANOVA). The rank-sum test was applied for irregular distributions or variances. In all tests, P < 0.05 was considered statistically significant.

## 3 Results

### 3.1 Characteristics of the subjects

This study included 30 healthy participants (HP) and 40 patients with oligoasthenospermia. Among the patients, 23 had oligospermia (IOA-1), 11 had oligoasthenospermia (IOA-3), and 6 had asthenospermia (IOA-2). Characteristics of the healthy individuals and patients are shown in **Supplementary Table 1**.

### 3.2 Microbiological characteristics of seminal plasma

**Figure 1:**
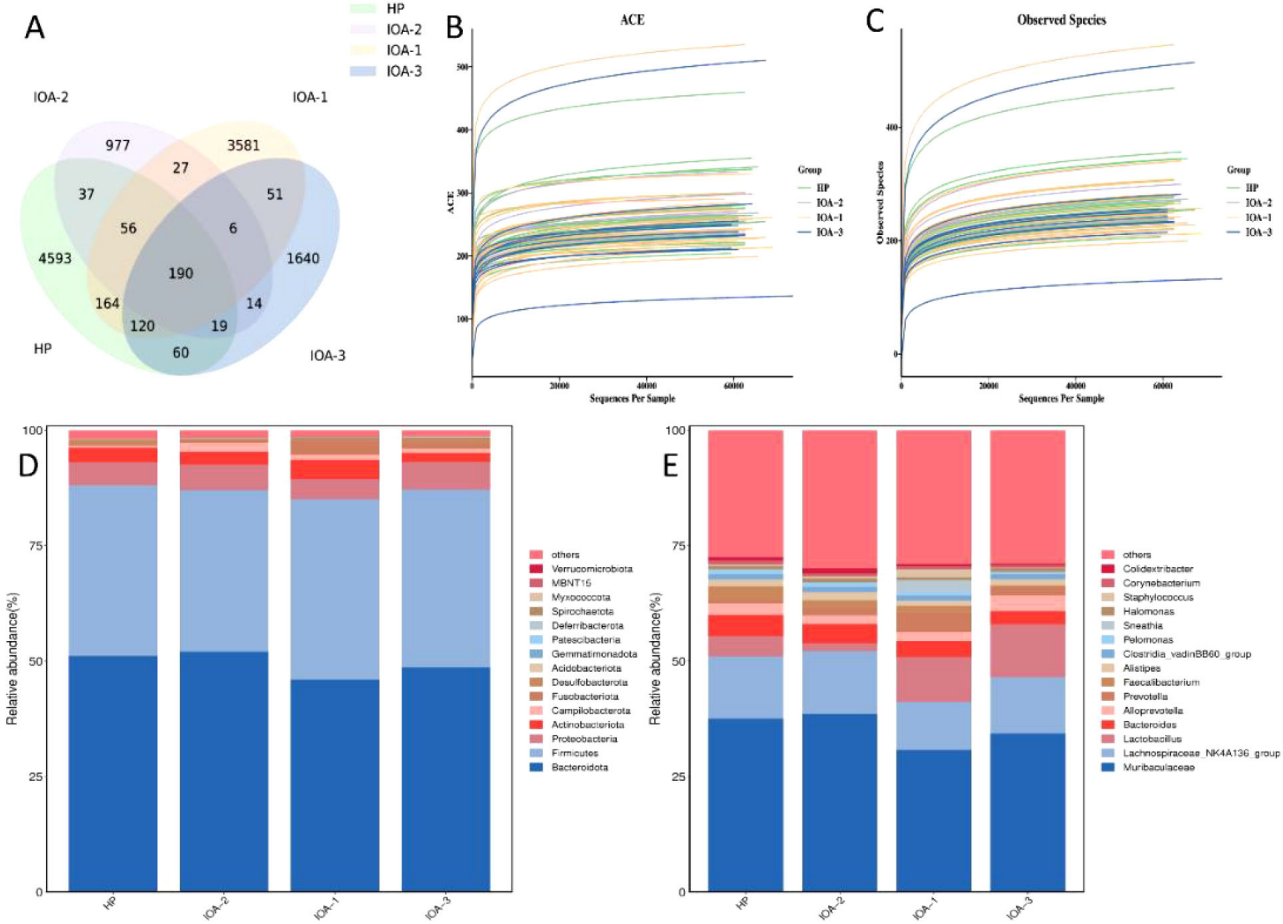
Results of 16S diversity sequencing analysis. **(A)** Venn diagram of sample ASVs statistics and distribution. **(B, C)** Dilution curve of ASV/ diversity index. **(D)** Proportion of categories according to the composition at the phylum level. **(E)** Proportion of categories according to the composition at the genus level.

This study included 70 samples analyzed *via* 16S rRNA gene targeted sequencing. After quality control and chimera removal, the valid tags (final data used for analysis) ranged from 58, 719 to 74, 285. A total of 11, 507 ASVs were identified across all 70 samples: The IOA-1 group yielded 4, 188 ASVs, with 3, 574 unique to this group. The IOA-2 group yielded 1, 326 ASVs, with 977 unique to this group. The IOA-3 group yielded 2, 093 ASVs, with 1, 633 unique to this group. The number of ASVs overlapping across all four groups was 190, accounting for approximately 1.6% of the total ASVs. ASV counts per sample ranged from 127 to 514 (**Figure 1A**). Species richness curves for all samples (**Figures 1B, C**) supported the adequacy of sampling.

The relative proportions of dominant taxa at the phylum and genus levels were assessed through the allocation of microbial taxonomic units. At the phylum level, the top four most abundant bacterial phyla were identical across the HP, IOA-1, IOA-2, and IOA-3 groups: *Bacteroidetes*, *Firmicutes*, *Proteobacteria*, and *Actinobacteria*. These four phyla accounted for over 90% of the total bacterial phyla in each group, with no significant differences in their relative abundance ratios among the groups (**Figure 1D**). At the genus level, *Muribaculaceae* and *Lachnospiraceae_NK4A136_group* ranked as the top two genera in relative abundance across all four groups, accounting for 40% to 50% of the total genus abundance in each group. Similarly, no significant differences in relative abundance were observed for these two genera among the groups (**Figure 1E**).

**Figure 2:**
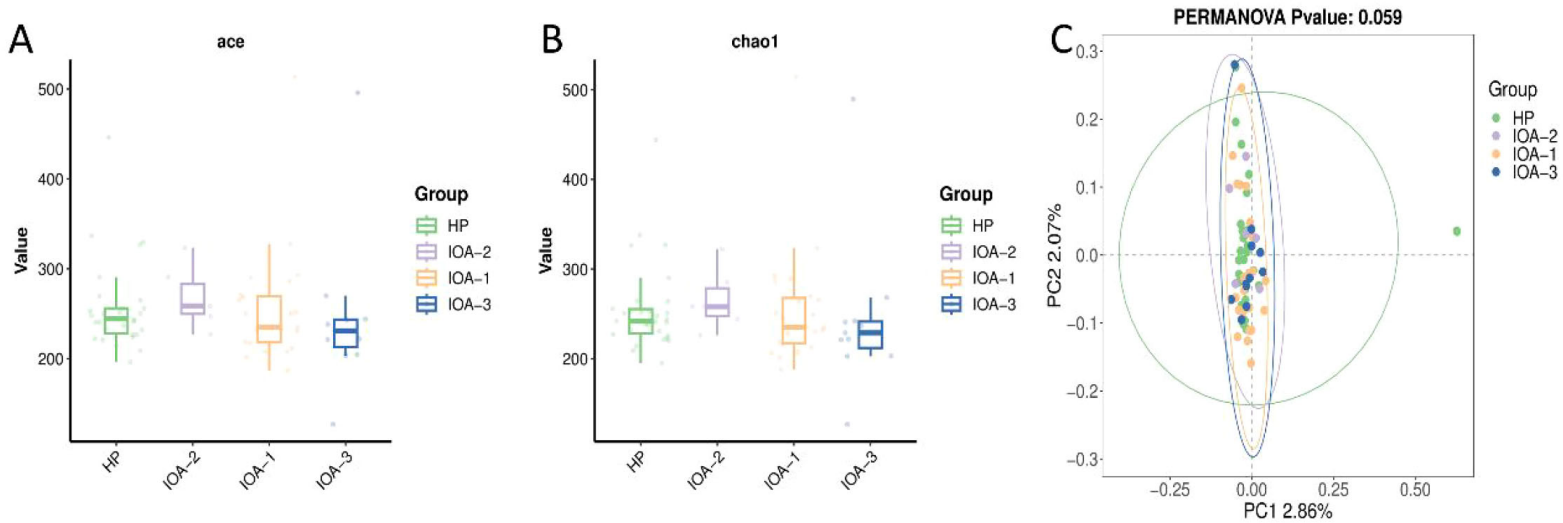
Analysis of semen microbiome diversity. **(A)** Boxplot analysis of alpha diversity correlation(Based on ACE index). **(B)** Boxplot analysis of alpha diversity correlation (Based on the Chao1 index) **(C)** Beta diversity analysis(PcoA plot based on Binary-Jaccard distance).

To assess differences in bacterial diversity among groups, sequences were aligned to estimate α-diversity and β-diversity. First, we used the Ace index and Chao1 index to evaluate species richness in human semen bacterial communities, where higher Ace and Chao1 indices indicate greater species richness. The study revealed that species richness among the four sample groups ranked from highest to lowest as follows: IOA-2 group > HP group > IOA-1 group > IOA-3 group (**Figures 2A, B**). Second, PcoA analysis based on Binary-Jaccard distance (**Figure 2C**) revealed some taxonomic differences among the four groups, but no statistically significant differences (P = 0.059, P>0.05).

**Figure 3:**
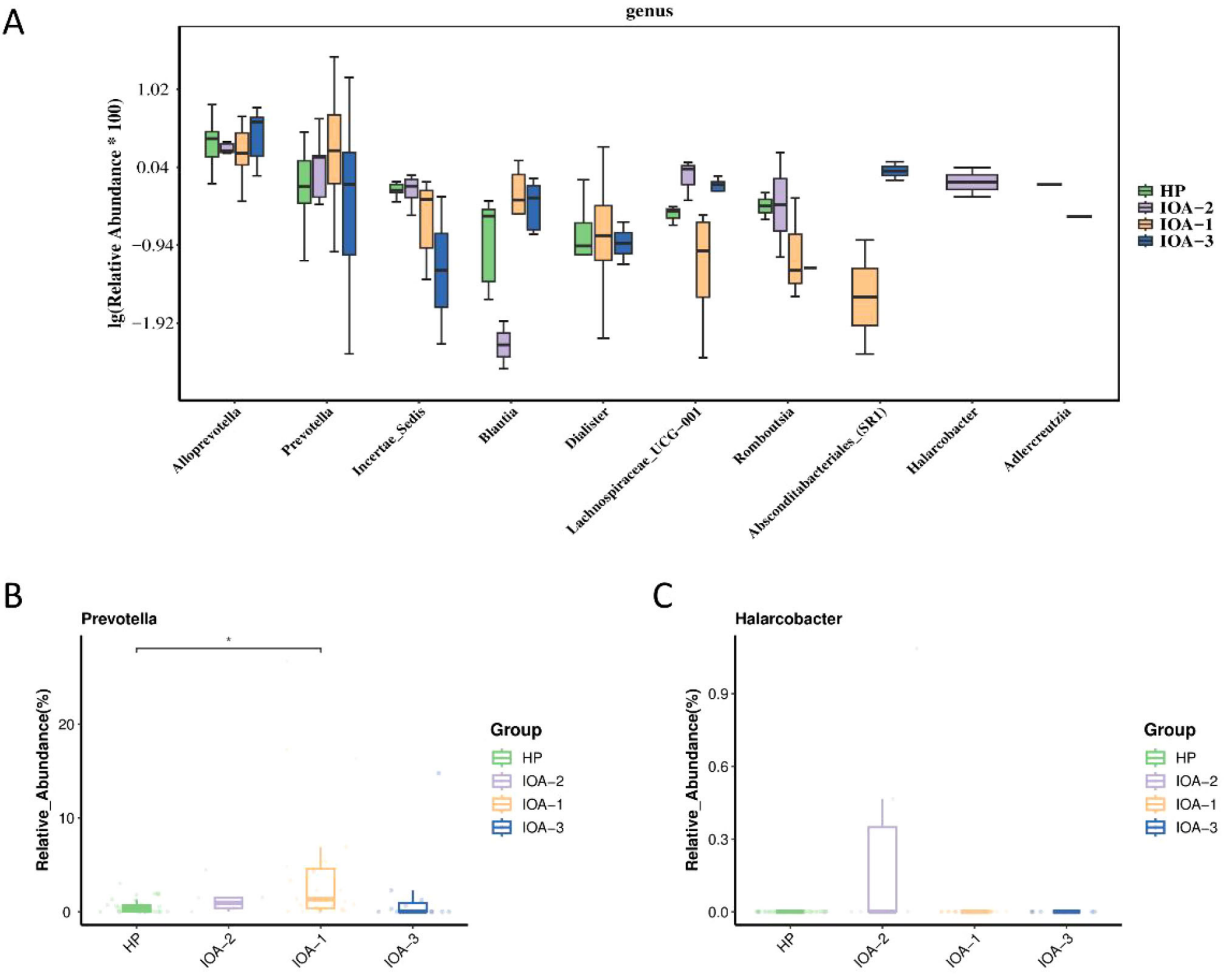
Top10 boxplot analysis of the abundance of different species. **(A)** Top10 boxplot of the abundance of different species. **(B, C)** Top10 boxplot of the abundance of different species in individual bacteria (Prevotella Halarcobacter). P-value, *p < 0.05; p < 0.01; *p < 0.001.

**Figure 4:**
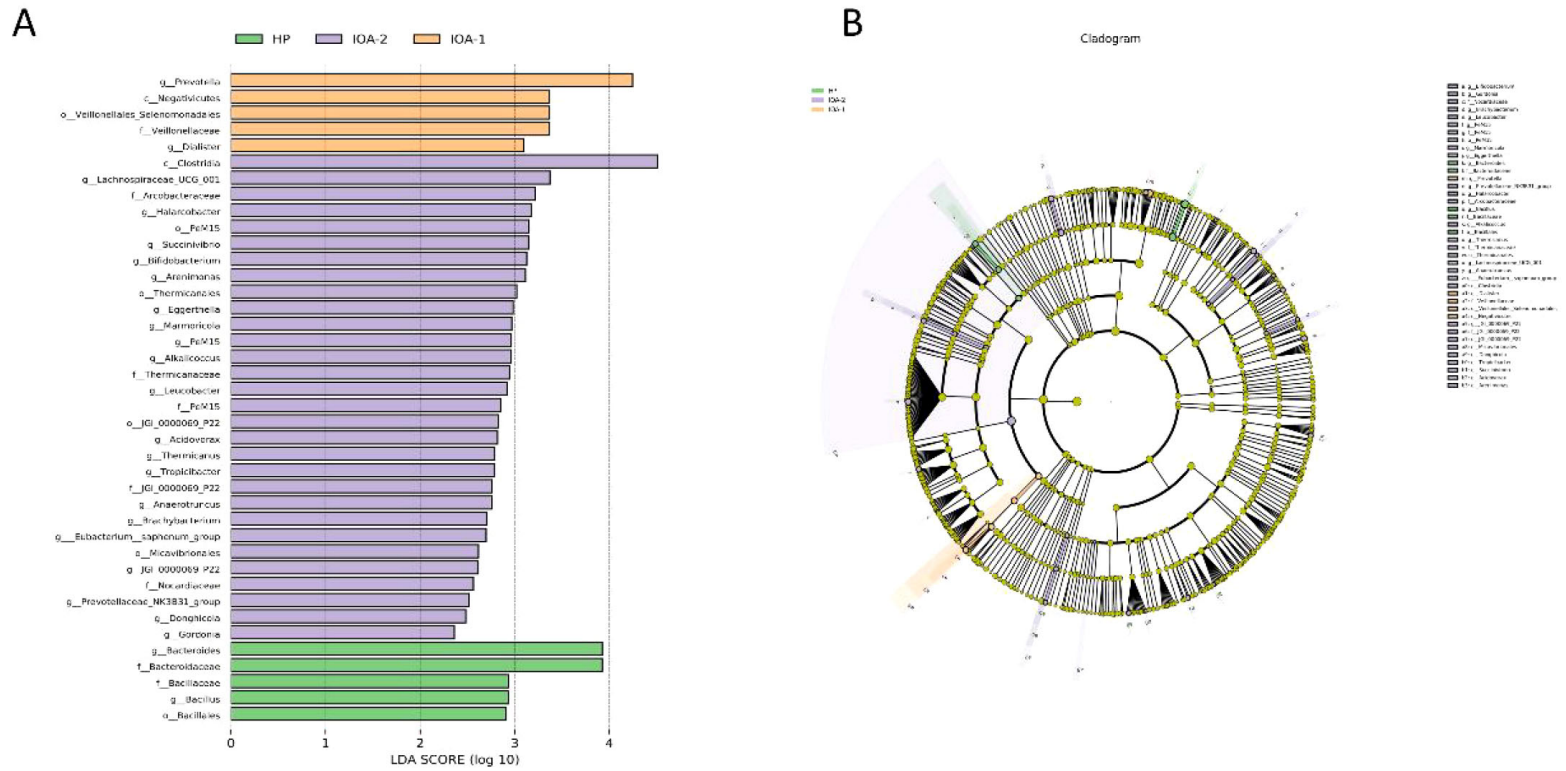
LEfSe analysis. **(A)** Histogram of LDA value distribution **(B)** cladogram.

We performed statistical analysis at the genus level, selecting the top 10 differentially abundant microbial species for boxplot analysis of relative abundance (**Figure 3A**). This revealed the abundance of dominant differential species within groups and between groups. Compared to the HP group, *Prevotella* abundance was significantly increased in the IOA-1, IOA-2, and IOA-3 groups (P = 0.036) (**Figure 3B**). Compared to the IOA-2 group, *Halarcobacter* abundance was significantly reduced in the HP, IOA-1, and IOA-3 groups (P = 0.0001) (**Figure 3C**). Finally, linear discriminant analysis with effect size measurement (LEfSe) was employed to generate differential species score plots and annotated analysis examples, identifying species with significant effects within each group to pinpoint bacteria associated with specific categories (**Figures 4A, B**). The study revealed that several opportunistic pathogens, including *Prevotella*, *Negativicutes*, *Veillonellales_Selenomonadales*, *Veillonellaceae*, and *Dialister*, were significantly overrepresented in the IOA-1 group (LDA score (log10) > 3.0). *Clostridia* constituted the most abundant microbial group in the IOA-2 group (LDA score (log10) >4.0), while *Bacteroides* and *Bacteroidaceae* were significantly enriched in the HP group (LDA score (log10) >3.0). These findings indicate that alterations in semen microbiota composition correlate with semen quality.

### 3.3 The metabolomic characteristics of seminal plasma

**Figure 5:**
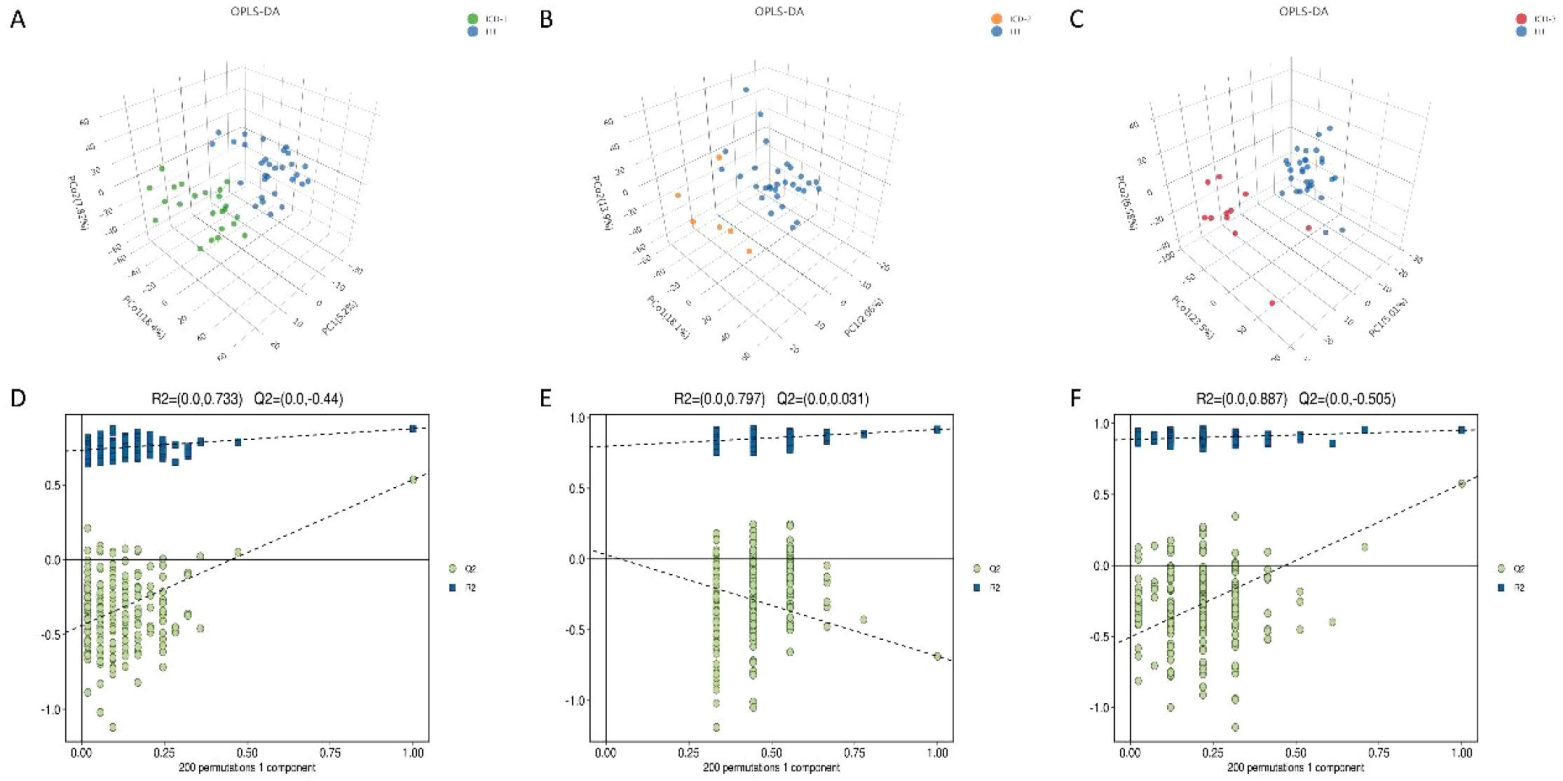
Multivariate statistical analysis of metabolomics. **(A-C)** Orthogonal partial least squares discriminant analysis (OPLS-DA) for comparison of metabolites in semen samples between different groups. **(D)** permutation diagram (HP vs IOA-1 ) **(E)** permutation diagram (HP vs IOA-2) **(F)** permutation diagram(HP vs IOA-3). mage caption: Model validity evaluation criteria:(1) All green Q2 values on the left are lower than the original point on the right.(2)The green regression line at Q2 intersects the vertical axis (left) at or below zero.

To investigate differences in seminal plasma metabolites among IOA patients, this study employed non-targeted metabolomics based on GC-MS and LC-MS technologies. First, supervised orthogonal partial least squares discriminant analysis (OPLS-DA) was used to enhance intergroup differential identification. The OPLS-DA model score plot demonstrated effective separation of principal components across groups (**Figures 5A-C**). Next, a permutation test (n=200) was performed to assess model overfitting and validate the reliability of the experimental statistical model. As shown, all blue Q2 values on the left side of the plots for both the IOA-1 vs. HP and IOA-3 vs. HP comparisons fell below the original points on the right, indicating the validity of the original models for these two groups (**Figures 5D, F**). However, the IOA-2 vs. HP model exhibited overfitting (**Figures 5E**), failing to distinguish groups effectively at the metabolic level. Therefore, we analyzed metabolite differences between the IOA-1 vs. HP and IOA-3 vs. HP groups.

**Figure 6:**
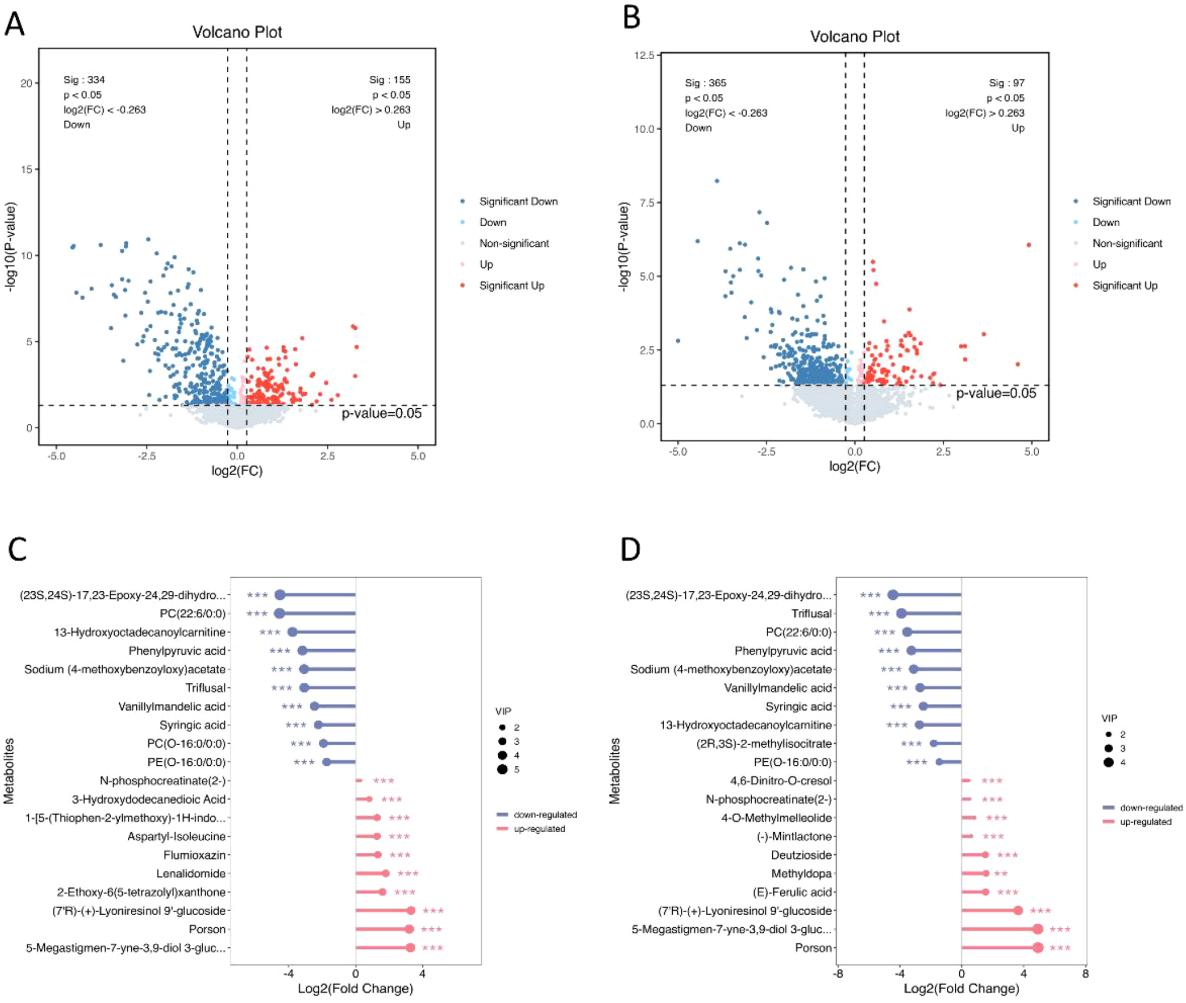
Identification of differential metabolites in seminal plasma. **(A)** Volcano plot (HP vs IOA-1). **(B)** Volcano plot (HP vs IOA-3). Volcano plots showing differentially accumulated metabolites in the disease group compared to the control group. Red dots indicate up-regulation of metabolites in the disease group, and blue dots indicate down-regulation of metabolites in the disease group. **(C)** Lolipopmap(HP vs IOA-1). **(D)** Lolipopmap(HP vs IOA-3). Red indicates up-regulation, blue indicates down-regulation, and asterisks indicate the significance of differential metabolism (P-value, *p < 0.05; **p < 0.01; ***p < 0.001.), and the dot size is determined by the VIP value.

Differential metabolite analysis was performed for each group using VIP > 1 combined with univariate analysis at P < 0.05. Compared with the HP group, the IOA-1 group identified 489 differential metabolites, including 155 upregulated and 334 downregulated metabolites (**Figure 6A**). The IOA-3 group identified 462 differential metabolites, comprising 97 upregulated and 365 downregulated metabolites (**Figure 6B**). The abundant differential metabolites primarily included phenolic compounds, lipids and lipoid molecules, organic acids and their derivatives, and organic oxygen compounds.

**Figure 7:**
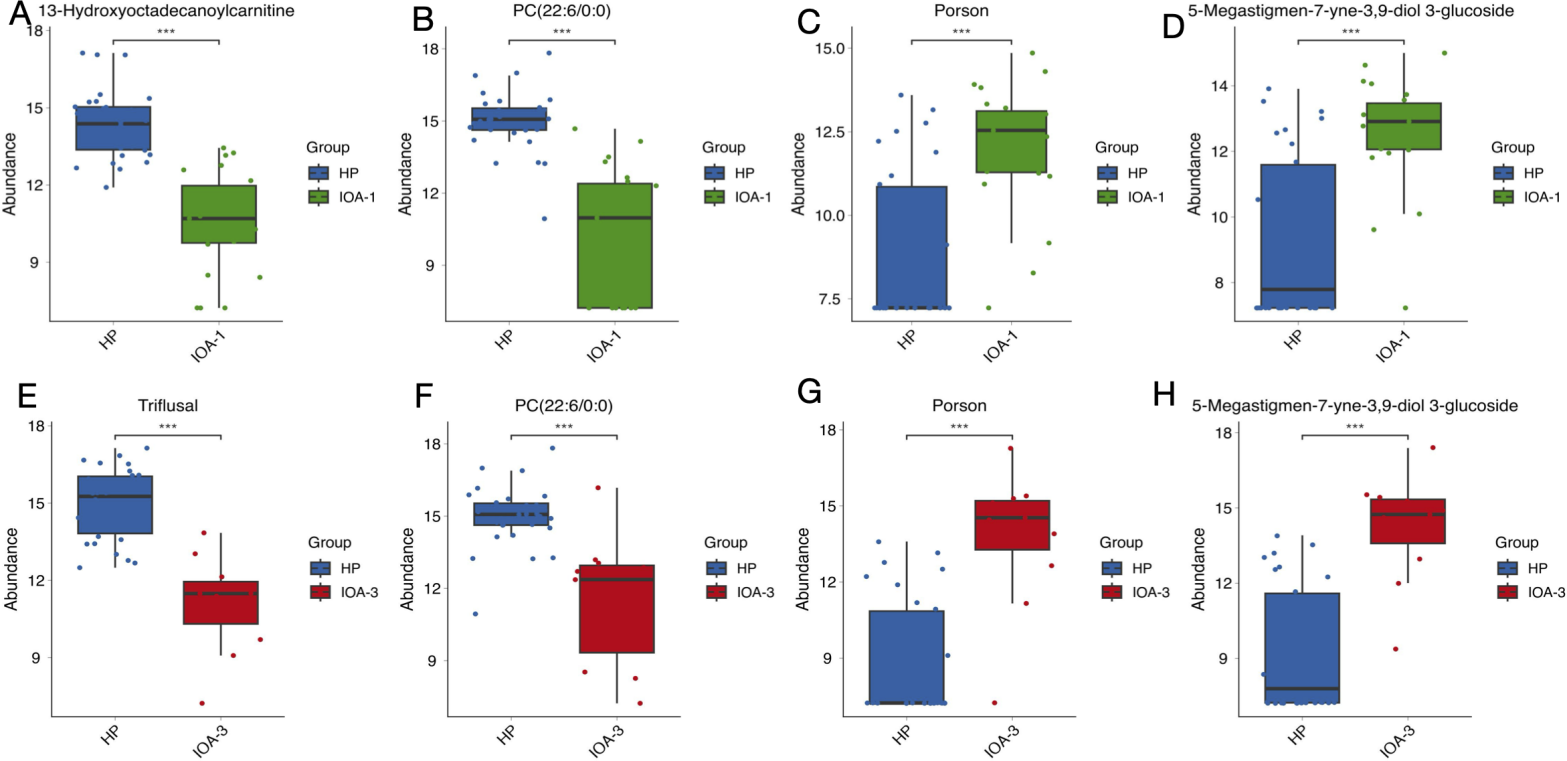
Distribution of significantly different metabolites between groups. **(A-H)** Box plots further illustrate differentially abundant metabolites with significant fold change in each comparison group. P-value, *p < 0.05; **p < 0.01; ***p < 0.001.

We then selected the top 10 differentially expressed metabolites with the smallest p-values from each comparison group. The study revealed that compared to the HP group, the IOA-1 group exhibited significant downregulation of 10 metabolites including Vanillylmandelic acid (P = 1.18E-11, FC = 0.18), Syringic acid (P = 7.73E-11, FC = 0.21), Phenylpyruvic acid (P = 5.50E-11, FC = 0.11), among others. In the IOA-1 group, Flumioxazin (P = 3.75E-05, FC = 2.51), Lenalidomide (P = 6.37E-06, FC = 3.48), and Porson (P = 1.32E-06, FC = 9.20) were significantly upregulated (**Figure 6C**). Among these, 13-Hydroxyoctadecanoylcarnitine (**Figure 7A**) and PC(22:6/0:0) (**Figure 7B**) exhibited expression levels more than 10-fold lower than in the HP group, while Porson (**Figure 7C**) and 5-Megastigmen-7-yne-3, 9-diol 3-glucoside (**Figure 7D**) showed nearly tenfold upregulation. In the IOA-3 group, metabolites including Triflusal (P = 5.85E-09, FC = 0.07), Vanillylmandelic acid (P = 6.75E-08, FC = 0.15), Syringic acid (P = 1.56E-07, FC = 0.18), and Phenylpyruvic acid (P = 7.63E-07, FC = 0.10) were significantly downregulated. Metabolites such as Porson (P = 8.51E-07, FC = 30), 5-Megastigmen-7-yne-3, 9-diol 3-glucoside (P = 8.758E-07, FC = 30.13), (E)-Ferulic acid (P = 0.00013, FC = 2.91) were significantly upregulated (**Figure 6D**). Among these, Triflusal (**Figures 7E**) and PC (22:6/0:0) (**Figure 7F**) showed more than 10-fold downregulation, while Porson (**Figures 7G**) and 5-Megastigmen-7-yne-3, 9-diol 3-glucoside (**Figure 7H**) exhibited 30-fold upregulation. Our data indicate that patients with oligoasthenozoospermia exhibit unique seminal plasma metabolites that distinguish them from healthy individuals.

**Figure 8:**
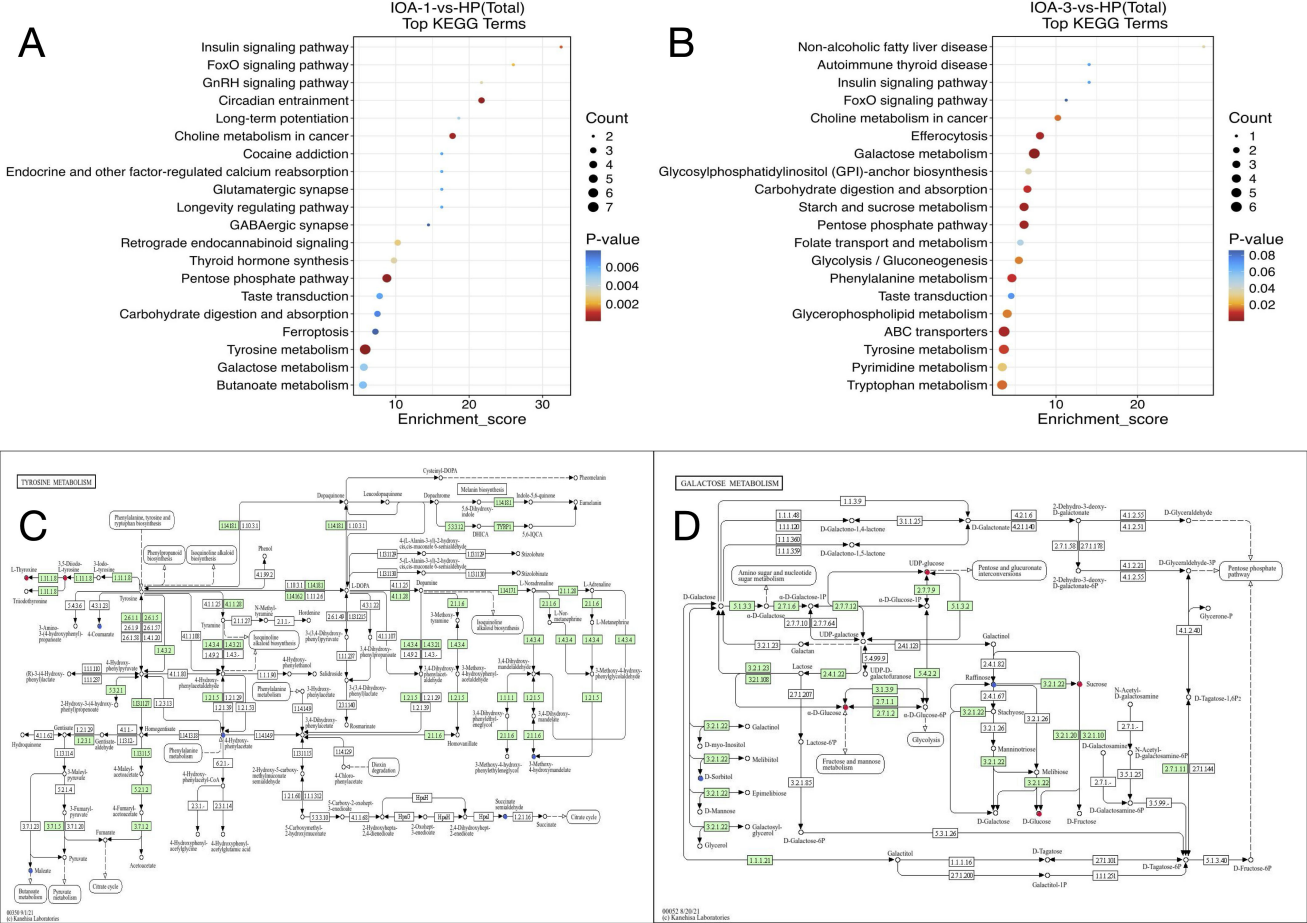
Pathway enrichment analysis. **(A)** Enriched top20 bubble map(IOA-1-vs-HP) **(B)** Enriched top20 bubble map (IOA-3-vs-HP). **(C)** KEGG network pathway map of Tyrosine metabolism. **(D)** KEGG network pathway map of Galactose metabolism. The small circles in the metabolic pathway diagram represent metabolites. The metabolites identified in red in the pathway map are the upregulated metabolites detected by the experiment, and the downregulated metabolites are shown in blue.

Next, we performed Kyoto Encyclopedia of Genes and Genomes (KEGG) pathway enrichment analysis on the differentially expressed metabolites to investigate the mechanisms underlying metabolic pathway changes in the differential samples. We selected the 20 pathways with the smallest p-values for significant enrichment among differentially expressed metabolites in each comparison group and visualized them in a bubble plot. Larger bubbles represent pathways containing more differentially expressed metabolites. Bubble colors transition from blue to red, with smaller p-values indicating greater significance. Compared to the HP group, 20 pathways were identified as key pathways associated with the IOA-1 group, including Tyrosine metabolism, Pentose phosphate pathway, Retrograde endocannabinoid signaling, and GnRH signaling pathway (**Figure 8A**). Among these, Tyrosine metabolism showed the most significant enrichment (p=0.0002, Count of differential metabolites=7, Enrichment_score=5.84). This pathway annotated seven differential metabolites: L-Thyroxine, 3, 5-Diiodo-L-tyrosine, 4-Coumarate, 4-Hydroxy-phenylacetate, Maleate, 3-Methoxy-4-hydroxymandelate, and Succinate semialdehyde (**Figure 8C**). The IOA-3 group showed significant enrichment in pathways including the pentose phosphate pathway, phenylalanine metabolism, tryptophan metabolism, and pyrimidine metabolism (**Figure 8B**). Among these, galactose metabolism (p=0.001, Count of differential metabolites=6, Enrichment_score=7.34) (**Figure 8D**) and ABC transporters (p=0.006, Count of differential metabolites=6, Enrichment_score=3.63) showed particularly high enrichment levels. The annotated differential metabolites in these two pathways were D-Sorbitol, a-D-Glucose, UDP-glucose, Raffinose, D-Glucose, Sucrose, Putrescine, Maltodextrin, Mannitol, a-Glucoside, N-Acetylglucosamine, and Glucose.

### 3.4 Microbiome-metabolome combined analysis

**Figure 9:**
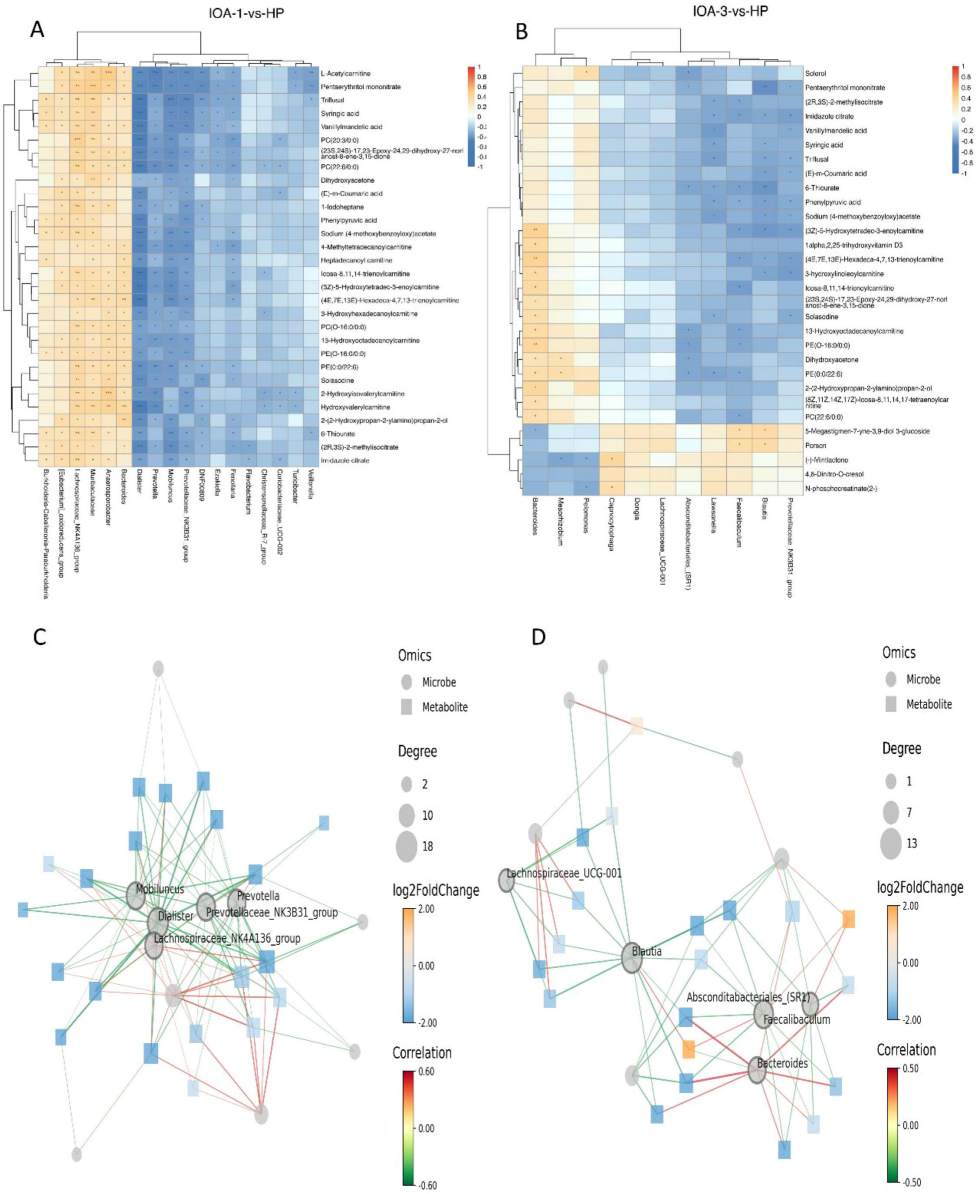
Cross-correlation analysis of microbiota and metabolites. **(A)** Correlation heatmap of top differentials(IOA-1-vs-HP). **(B)** Correlation heatmap of top differentials(IOA-3-vs-HP). **(C)** Graph of the correlation network of top differentials(IOA-1-vs-HP). **(D)** Graph of the correlation network of top differentials(IOA-3-vs-HP).

To investigate the potential mechanisms linking changes in semen microbiota and functional metabolites to disease progression, we performed a comprehensive network analysis integrating microbiome (genus-level) and metabolomics data using Spearman correlation algorithms. The top 30 significantly different entries (ranked by p-value) from both microbiome and metabolome datasets were included in the analysis. The correlation heatmap is shown in the figure. The results reveal complex co-occurrence patterns and strong correlations between differential semen microorganisms and metabolites. In the HP and IOA-1 comparison groups, the microorganism *Dialister* showed strong negative correlations with metabolites L-Acetylcarnitine, Syringic acid, PC(20:3/0:0), and PC(22:6/0:0) (**Figure 9A**). In the HP and IOA-3 groups, *Bacteroides* showed positive correlations with 1alpha, 2, 25-trihydroxyvitamin D3, PE(O-16:0/0:0), Dihydroxyacetone, PE(0:0/22:6), and PC(22:6/0:0) (**Figure 9B**). To better elucidate the potential intrinsic relationship between changes in semen microbial community structure and metabolite differences, a correlation network diagram was constructed based on the top 20 significantly different entries across each omics. Node connectivity was defined as the number of significant associations between the microorganism and metabolites, reflecting its centrality in microbe-metabolite interactions. Analysis revealed that in the HP versus IOA-1 comparison group, the microbes *Dialister*, *Prevotellaceae_NK3B31_group*, *Mobiluncus*, *Prevotella*, and *Lachnospiraceae_NK4A136_group* occupied core network nodes (**Figure 9C**). In the HP versus IOA-3 comparison, microorganisms *Lawsonella*, *Blautia*, *Prevotellaceae_NK3B31_group*, *Faecalibaculum*, and *Bacteroides* formed the core network nodes (**Figure 9D**), suggesting they may serve as key mediators in the regulation of host metabolism by the microbiome.

## 4 Discussion

In the post-genomic era, advanced multi-omics and digital approaches enable accurate diagnosis and molecular stratification of deteriorating semen quality and idiopathic male infertility (Assidi, 2022). We investigated the network associations between the semen microbiome, metabolome, and idiopathic oligoasthenozoospermia. We found significant alterations in the diversity and composition of the semen microbiome and metabolome in patients with idiopathic oligoasthenospermia.These changes may impact sperm quality through pathways such as inducing oxidative stress, stimulating excessive inflammation, and disrupting material and energy metabolism essential for spermatogenesis. Further analysis suggests that combined assessment of the semen microbiome and metabolites may serve as a non-invasive biomarker for diagnosing idiopathic oligoasthenospermia. To our knowledge, this represents the first detailed characterization of the semen microbiome-metabolite-host relationship in idiopathic oligoasthenospermia.

The alpha diversity of seminal plasma microorganisms in the IOA-2 group ranked first among the four groups. This might be due to the specific imbalance of the seminal plasma microecology. Compared with the healthy group, the sperm motility in this group decreased without a clear cause, accompanied by changes in key microorganisms such as *Bacteroides*, which disrupted the stable structure of the normal flora and created conditions for the colonization of various microorganisms. Compared with the IOA-1 group and the IOA-3 group, the damage to spermatogenesis function in the latter two groups would severely disrupt the nutritional supply of seminal plasma and the stability of the microenvironment, making it difficult for the microbiota to diversify and survive, often showing a trend of single-bacterial dominance or overall reduction in the abundance of the microbiota. However, the IOA-2 group did not experience such a severe microenvironment collapse, thus presenting the highest alphadiversity (Garcia-Segura et al., 2022).We found that *Bacteroidetes*, *Firmicutes*, *Proteobacteria*, and *Actinobacteria* were the top four phyla in the semen microbiome, consistent with the findings of Fu et al (Fu et al., 2024). *Bacteroidetes* and *Firmicutes* are known to be dominant phyla in the human gut microbiota, and the *Firmicutes*-to-*Bacteroidetes* ratio is considered closely associated with age and the occurrence of certain diseases (Mariat et al., 2009; Bahar-Tokman et al., 2022; Takezawa et al., 2021).Research indicates that male azoospermia is associated with reduced *Bacteroidetes* abundance (Alfano et al., 2018), while patients with leukospermia exhibit a decreased *Firmicutes*/*Bacteroidetes* ratio in their semen microbiota (Yao et al., 2022). Furthermore, we observed a significant increase in *Prevotella* abundance within the semen microbiome of infertile males. *Prevotella* has been demonstrated to be associated with diminished male semen quality (Farahani et al., 2021). Known as a Gram-negative obligate anaerobe, *Prevotella* has evolved multiple virulence factors including adhesins, hemolysins, extracellular polysaccharides, lipopolysaccharides (LPS), proteases, and quorum-sensing molecules, thereby evolving into highly adaptive pathogens capable of successful infection and proliferation within host tissues (Sharma et al., 2022). Furthermore, our LEfSe analysis confirmed *Prevotella* as a specific bacterial indicator for identifying IOA-1. Furthermore, *Clostridia*, as opportunistic pathogens with significant roles in IOA-2, have been previously identified in studies on boar semen microbiome and reproductive potential (Pinart et al., 2017; Gòdia et al., 2020; Bonet et al., 2018).The presence of *Clostridium* species as pathogens within the semen microbiome has been associated with reduced sperm motility, increased sperm agglutination, and damage to sperm cell membranes. *Clostridia* are thought to alter sperm quality by secreting endotoxins or adhering to sperm cell membranes, causing physiological changes in sperm cells (Köhn et al., 1998; Petit et al., 1999). *Bacteroides* and *Bacteroidaceae* contribute most significantly to HP group differences. Studies indicate that *Bacteroides* constitute 20% to 80% of the gut microbiota. A diverse *Bacteroides* population helps balance the gut microbiome, prevent pathogenic overgrowth, and maintain a stable microbial ecosystem (Tufail and Schmitz, 2025; Gámez-Macías et al., 2024). They promote the production of short-chain fatty acids (SCFAs) like butyrate, which maintain the intestinal barrier and exert anti-inflammatory effects. Concurrently, these bacteria participate in regulating the host immune system by influencing immune cell maturation and cytokine production. This regulation is crucial for immune tolerance and pathogen prevention (Magalhães et al., 2025).

In studies of seminal plasma metabolomics, significant downregulation was observed in the expression levels of 13-Hydroxyoctadecanoylcarnitine and 1-Palmitoyl-2-docosahexaenoyl-sn-glycero-3-phosphocholine (PC(22:6/0:0)) within the IOA-1 group. Research indicates that carnitine exhibits the highest efficacy among all antioxidants. Within human tissues, carnitine scavenges superoxide anion and hydrogen peroxide radicals, thereby protecting sperm cells from oxidative damage and improving sperm quality (Su et al., 2022). Additionally, L-carnitine exhibits anti-apoptotic effects on germ cells, inhibiting programmed cell death mediated by the FAS-FAS ligand pathway and caspases 3, 7, and 8 (Mongioi et al., 2016; Mutomba et al., 2000). Docosahexaenoic acid (DHA; 22:6 n-3), an omega-3 polyunsaturated fatty acid, is a potentially important antioxidant with good safety profiles. It improves the body's antioxidant status, thereby reducing sperm DNA fragmentation (Martínez-Soto et al., 2013, Martínez-Soto et al., 2016).KEGG enrichment analysis revealed that the most significant enrichment of differential metabolites between the IOA-1 group and the HP group occurred in Tyrosine metabolism. Dopamine, norepinephrine, and other catecholamines produced *via* Tyrosine metabolism are known to regulate the function of the hypothalamic-pituitary-gonadal (HPG) axis (Hase et al., 2015; Kaiser and Jaillardon, 2023), thereby affecting testosterone secretion. Testosterone is a core hormone for maintaining spermatogenesis, and abnormal testosterone levels directly lead to reduced sperm count and decreased motility. Disturbances in Tyrosine metabolism ultimately impact sperm quality. This study found that differential metabolites between the IOA-3 group and the HP group showed significant enrichment in Galactose metabolism and ABC transporters. The enrichment in Galactose metabolism aligns with previous targeted metabolomics studies on cryopreserved sperm. Sperm freeze-thaw processes induce significant declines in sperm motility and alterations in other parameters: membrane and acrosome integrity, DNA fragmentation index (DFI), and reactive oxygen species (ROS). This process involves substantial changes in multiple pathways, including Galactose metabolism (Raad et al., 2018; Fu et al., 2019). This pathway is known as a key branch of human carbohydrate metabolism, regulating the conversion of galactose to glucose while participating in the glycosylation modification of biomolecules such as glycoproteins and glycolipids. These modified products are crucial for maintaining cellular structural stability and signal transduction (Coelho et al., 2015). Abnormalities in this pathway can lead to excessive galactose accumulation, causing oxidative damage to sperm membranes or impairing the synthesis of sperm surface glycoproteins. Ultimately, this affects sperm quality and the process of sperm-egg binding (Fan et al., 2024; Lahnsteiner et al., 2010). As a core pathway for transmembrane transport, ABC transporters form a critical transport network in testicular supporting cells, serving as the cornerstone of blood-testis barrier function and determining the foundation of spermatogenesis (Ribeiro et al., 2024). Additionally, this pathway enhances antioxidant capacity by facilitating the transport of antioxidants such as vitamin E and glutathione into sperm, thereby safeguarding sperm motility and fertilization potential (Gregory and Cyr, 2014).

Comprehensive network analysis revealed that in the HP and IOA-1 comparison groups, the microorganism *Dialister* exhibited strong negative correlations with four metabolites. L-Acetylcarnitine is known as the core energy carrier for mitochondrial β-oxidation in sperm, directly determining ATP production efficiency (Khaw et al., 2020). Syringic acid scavenges reactive oxygen species (ROS) and protects sperm DNA (Akarsu et al., 2024). PC(20:3/0:0) and PC(22:6/0:0) are core components of the sperm membrane, maintaining membrane fluidity and acrosome reaction capacity (Yang et al., 2020). This finding reveals the association between *Dialister* in semen and key metabolites related to “energy metabolism-antioxidation-membrane structure, ” suggesting that excessive proliferation of *Dialister* may be a potential driver of declining sperm quality. In the HP and IOA-3 groups, *Bacteroides* showed strong positive correlations with certain metabolites. Among these, 1α, 2, 25-trihydroxyvitamin D3 is a key active form of vitamin D involved in immune regulation and cellular metabolism (Kumagai et al., 2012), while phospholipids (PE, PC) are essential components of cell membranes (Yang et al., 2020; Santos et al., 2024). It is speculated that Bacteroides may regulate vitamin D metabolism and cell membrane lipid homeostasis in semen; however, the specific direction of regulation and underlying mechanisms require further validation.

Our study has several limitations. First, as a single-center design with a limited sample size, selection bias may exist, potentially restricting the external generalizability of our conclusions. For instance, in the metabolomics analysis, there was overfitting between the IOA-2 group and the HP group models. Therefore, we were unable to conduct a comparative analysis at the metabolomics level between the two groups. We speculate that the insufficient sample size might be the primary reason for this.Future multi-center, large-scale studies are needed to validate these findings. Second, 16S rRNA gene sequencing only enables microbial taxonomic identification and struggles to deeply analyze microbial functions, potentially overlooking critical functional associations. Furthermore, metabolomics analysis identified disease-associated differential metabolites but did not establish causal relationships between these metabolites and sperm count/motility. The specific regulatory mechanisms require further mechanistic experimentation. Finally, the study lacks longitudinal follow-up data, preventing clarification of the dynamic changes in the semen microbiome and metabolome and their impact on long-term reproductive outcomes.

## Data Availability

The original data of the microbial group sequencing reported in this article has been stored in the National Genomics Data Center (Nucleic Acid Research 2025), and the Genomic Sequence Archive of the Chinese Academy of Sciences/Biological Information Center/Beijing Genomics Institute (GSA: CRA035998) (Genomics, Proteomics and Bioinformatics 2025), and can be accessed publicly at https://ngdc.cncb.ac.cn/gsa; The metabolomics data reported in the article has been stored at the National Bioinformatics Center of the Chinese Academy of Sciences/Beijing Institute of Genomics (OMIX, https://ngdc.cncb.ac.cn/omix: Deposit number OMIX013948).
