# Supplementary material for: Comprehensive microbiome and metabolome analysis revealed the changes of semen microbial characteristics and metabolic phenotypes in patients with idiopathic oligoasthenozoospermia
Source: Front Cell Infect Microbiol. 2026 Jan 14;15:1741184. doi: 10.3389/fcimb.2025.1741184 (PMC12847254; doi:10.3389/fcimb.2025.1741184)
Supplement: Supplementary file 1 [file Table1.docx]

Supplementary Table 1:Basic information for participants

| **Characteristics** | **Control group（n=30）** | **Oligospermia group（n=23）** | **Asthenospermia group（n=6）** | **Oligoasthenospermia**  **group（n=11）** | **p-value** |
| --- | --- | --- | --- | --- | --- |
| Age（years） | 30.70±5.43 | 32.13±4.48 | 32.33±7.34 | 33.55±5.40 | 0.489 |
| BMI (kg/m2) | 24.93±3.78 | 24.29±2.52 | 23.02±1.09 | 25.23±3.32 | 0.513 |
| SMR (%) | 65.07±12.07 | 55.48±11.65 | 23.17±7.86 | 26.73±15.95 | / |
| PR (%) | 56.23±10.93 | 47.96±10.91 | 18.33±5.96 | 16.71±11.39 | / |
| SC（*10^6^/ml） | 89.73±49.29 | 6.55±3.99 | 74.23±36.37 | 6.20±4.97 | / |
| TSC(*10^6^) | 263.44±180.70 | 22.79±19.39 | 211.18±89.67 | 15.87±12.07 | / |

Abbreviations: BMI body mass index ,SMR Sperm motility rate, PR Sperm concentration, SC Sperm concentration, TSC Total sperm count
